# Beyond Sugar: A Holistic Review of Sweeteners and Their Role in Modern Nutrition

**DOI:** 10.3390/foods14183182

**Published:** 2025-09-12

**Authors:** Nela Dragomir, Daniela-Mihaela Grigore, Elena Narcisa Pogurschi

**Affiliations:** Faculty of Animal Productions Engineering and Management, University of Agronomic Sciences and Veterinary Medicine of Bucharest, 59 Marasti Blvd, District 1, 011464 Bucharest, Romania; nela.dragomir@usamv.ro (N.D.); elena.pogurschi@usamv.ro (E.N.P.)

**Keywords:** sugar, artificial sweetener, natural sweetener, nutrition, diabetic

## Abstract

This manuscript provides an in-depth review of both artificial and natural sweeteners, including polyols and plant-derived alternatives, examining their sweetening potency, glycemic index, modes of action, and applications in the food sector. The rising demand for sugar substitutes, fueled by health concerns such as obesity and diabetes, has prompted significant research into low-calorie and non-nutritive sweeteners. This work categorizes sweeteners into synthetic options (such as aspartame, sucralose, saccharin) and naturally occurring ones (such as stevia, monk fruit, and polyols like sorbitol, xylitol, erythritol), focusing on physico-chemical characteristics, relative sweetness (ranging from 100 to 220,000 times sweeter than sucrose), and glycemic index, important for their use in diabetes-friendly food products. The current manuscript examines how these sweeteners interact with taste receptors to induce sweetness perception without contributing significant calories. It also discusses their health implications and controversies and limitations regarding healthy and safety data, process feasibility, market application trends, environmental stability, and commercialization challenges. The review also addresses challenges in scaling production and ensuring the economic viability of plant-based sweeteners, offering a forward-looking perspective on their commercialization in the food industry.

## 1. Introduction

The sugar-reduction trend has become increasingly popular among consumers, and food companies have a duty to explore new ways to balance the scales of consumers interested in indulgence with better-for-you varieties [1]. Table 1 shows a list of sugars and caloric sweeteners commonly used as alternatives for sweetening food products. Sugar is a ubiquitous ingredient in food products [2], both for the sweet taste and for the technological and sensory effects it imparts to them [3]. As far as pastries are concerned, today’s society’s concerns are aimed at achieving a healthier result without sacrificing the flavor of a dessert. Traditionally, a pastry is distinguished by two key elements: sweetness and fat content [4,5,6]. The paradox arises when precisely these two elements have to be reduced or replaced with ingredients that have the same taste profile and ingredients that consumers want [7]. Therefore, reducing sugar content without compromising sensory attributes requires careful consideration of appropriate reduction techniques and the incorporation of suitable sugar alternatives.

**Table 1:** Common sweeteners and their Glycemic Index (GI).

Mono- and Disaccharides	Type of Treatments	Chemical Formulas	Glycemic Index (GI)	Potency *	References
Maltose	enzyme-catalyzed hydrolysis	C_12_H_22_O_11_	105	0.3	[8]
Dextrose	hydrolysis	C_6_H_12_O_6_	100	0.7	[9]
Glucose	hydrolysis	C_6_H_12_O_6_	100		[10]
Trehalose	enzymatic and/or fermentation processes	C_12_H_22_O_11_	70	0.4–0.5	[11]
Sucrose	extracted and purified through a series of physical and chemical operations	C_12_H_22_O_11_	65	1.0	[12]
Fructose	enzymatic conversion of glucose	C_6_H_12_O_6_	23	1.17–1.75	[13]
Galactose	hydrolysis of lactose or microbial fermentation	C_6_H_12_O_6_	20	0.5–0.7	[14]
Lactose	concentration, crystallization, and purification from dairy	C_12_H_22_O_11_	46	0.2–0.4	[15]
Tagatose	isomerization of D-galactose	C_6_H_12_O_6_	1–3	0.9	[16]
Isomaltose	starch hydrolyzation	C_12_H_22_O_11_	35	3.5	[17]

* Approximate potencies of high-potency sweeteners (sucrose = 1.0).

Replacing or reducing sugar in recipes can be accomplished with a variety of alternatives [18]. However, in choosing sugar alternatives, we must take into account consumer preferences [19], which tend to categorize natural ingredients as healthy and synthetic ingredients as unhealthy [20].

Chronic overconsumption of added sugars, especially from sugar-sweetened beverages, has been strongly linked to obesity, type 2 diabetes, cardiovascular disease, non-alcoholic fatty liver disease, kidney disease, gout, and tooth decay, with emerging evidence also suggesting possible connections to certain cancers and cognitive decline [21,22,23,24]. Potential harms are driven by mechanisms such as insulin resistance, liver fat accumulation, increased triglycerides, uric acid elevation, and enamel erosion, making excess sugar a major contributor to global health burdens [25,26]. Mitigating the health risks, reducing sugar intake is a public health priority, and alternatives like non-nutritional sweeteners (NNS) have gained attention as tools to lower sugar consumption without sacrificing taste [27]. Currently, the consumers are exposed to an overwhelming influx of information from popular media and various other sources, a substantial proportion of which is inaccurate or misleading. The consumer needs to understand that natural or synthetic sugar alternatives are checked [28], analyzed [29], studied and then given access to be added to human or animal food [30]. The sweeteners have been thoroughly studied and rigorously evaluated by experts, and their use in food is governed by explicit regulations [31,32,33]. The initiative to reduce or replace sugar in foods comes as a recommendation for the negative impact of excessive sugar consumption on public health [34]. The WHO guidelines recommend a free sugar intake of 10% of total daily energy intake throughout life [35].

Sweet taste has a powerful effect on the sensation of pleasure. Sweet taste receptors are the taste buds located on the surface of the tongue, which allow molecules and ions taken up from food to reach the receptor cells inside [36]. Sweet and umami are the main tastes that give this sensation of pleasure [37]. Sweet-tasting foods have led to consumer demand for savory foods and a large-scale production of caloric sweetening agents such as sucrose and fructose. However, the high demand for sugar and the succession of economic events that initially made this ingredient unavailable prompted the development of new alternative sweetening ingredients. The NNS were the first synthetic alternatives to sugar to be used in the development of novel foods [38]. Since then, the market has developed and evolved and today we have a wide range of natural, synthetic, and semi-synthetic sweeteners with negligible caloric contribution and which provide the ideal sweet taste [39,40].

Beyond their role in providing sweetness, sugars and sweeteners also influence metabolic responses, which can be evaluated through the concept of the glycemic index (GI). The GI represents a carbohydrate food’s relative ability to increase glucose in the blood. The human body needs glucose for energy, activity, and brain function. It is supplied to the body by the food ingested during the course of a day [41]. The metabolic process carried out by the body through the digestion of carbohydrate-containing foods determines the concentration of glucose in the blood, which is quantified by a parameter called blood glucose. A blood glucose level oscillating at extreme values indicates an imbalance in the body in terms of the amount of glucose. This leads to two situations: a drop in blood glucose (hypoglycemia) and an increase in blood glucose above normal levels (hyperglycemia) [42]. Both situations occur due to the amount of sugar provided by the foods in the daily diet.

The GI parameter divides foods on a 0–100 scale according to their effect on blood glucose levels [43]. There are 3 glycemic ranges that include: low GI (less than 55), medium GI (55 to 70), and high GI (greater than 70) [44]. Depending on the high-GI foods are digested and absorbed quickly, and blood glucose levels rise rapidly, and low-GI foods are digested more slowly, helping to maintain an even blood glucose level [45].

## 2. Artificial Sweeteners and Other Sugar Substitutes

Sweeteners are substances that provide sweetness, are approved for consumption, and have variable taste profiles (clean or with an aftertaste) and are approved for use in the human diet [46,47]. The stability and performance of sweeteners can be influenced by the type of processing or the presence of other ingredients, making some unsuitable for baking or freezing [48,49]. Sweetening power is defined as the ratio between the concentration of sucrose in a given medium and the concentration of sweetener that develops a sweet taste of the same intensity [50]. Compared to sugar, NNS have fewer or zero calories [51].

There are two very different categories of sweeteners: natural sweeteners: these are polyols (maltitol, xylitol) and plant-based (stevia, monk fruit); synthetic sweeteners have a much higher sweetening power than sucrose [52]. Artificial sweeteners are highly sweetening substances that contain no calories or carbohydrates and do not increase blood glucose levels [53]. Each artificial sweetener is recommended for a narrow range of foods or uses [54]. Some are recommended to be used only in cold drinks or preparations, and others in food processing or cooking, where hot processing is used [55]. Table 2 shows the synthetic sweeteners most commonly used in confectionery.

The metabolism of sweeteners differs from that of sugar in that they elicit little or no insulin secretion. Therefore, in both types of diabetes, levels of needed insulin must be adjusted if the sweetener is caloric (such as sugar alcohols) [10,56,57].

**Table 2:** Artificial sweeteners potency.

Sweeteners ^1^	Potency *	References
Advantame	20,000	[10]
Acesulfame-K	200	[58]
Alitame	2000	[59]
Aspartame	180	[60]
Cyclamate	30–40	[61]
Neotame	8000	[62]
Neohesperidin dihidrochalcon (NHDC)	1500–1800	[63]
Saccharin	300	[64]
Sucralose	600	[65]
Lugduname	220,000	[66]

* Approximate potencies of high-potency sweeteners (sucrose = 1.0); ^1^ all sweeteners are 0 kcal/g, except for aspartame, 4 kcal/g.

Sugar reduction is an ongoing consumer trend affecting the bakery industry [48,49]. However, replacing sugar is difficult because of the property’s sucrose exhibits during baking [50]. Moreover, the concept of sweetening power has certain limitations due to the fact that they are linked to the perception of sweetness, a subjective aspect that differs from one individual to another [49]. Each sweetener has its own sensory characteristics; there are no sweeteners with a taste profile identical to that of sucrose, and assessing a sweetener solely on the basis of sweetness is incorrect [52].

### 2.1. Saccharin

Saccharin is a white, crystalline, colorless powder. The sweet taste of saccharin develops slowly, but after reaching its maximum intensity, it is quite persistent [67]. Saccharin has a residual bitter/metallic aftertaste, which becomes very strong at high concentrations. It is also marketed in pill form with 500 times the sweetening power of sugar [68]. It is soluble in water and stable at high temperatures (one hour at 150 °C) and pH variations (2–7), which makes it suitable for use in a wide range of foods [69,70]. It is less commonly used as such because of its bitter-metallic taste. To mitigate the effects of this negative aspect, saccharin is used in mixtures with other sweeteners that mask or mitigate the metallic taste (polyols, fructose, cyclamates, aspartame) [71]. It is therefore frequently used in mixtures with synergistic action: cyclamates:saccharin (10:1), fructose:saccharin, or aspartame:saccharin (2:1) [72]. Saccharin is not recommended for addition to recipes for baked products because it is unstable at high temperatures and develops a bitter taste [73]. Saccharin is absorbed slowly in the human intestine, and once in the bloodstream, it is rapidly eliminated as such and not metabolized [74]. Currently, saccharin is considered a substance of low toxicity, with an acceptable daily intake of 0–5 mg/kg body weight, according to the FAO [75].

### 2.2. Aspartame

Aspartame was discovered in 1965, and in 1981 it was approved in over 100 countries for current consumption (in cereals, chewing gum, gelatine, pudding, and soft drinks) [71]. Aspartame is a combination of two amino acids, L-aspartic acid and L-phenylalanine [76]. The presence of L-phenylalanine in the formula prohibits consumption for people with phenylketonuria [77]. It occurs as a white crystalline powder or in granular form, soluble in water and ethyl alcohol [78]. Aspartame breaks down rapidly under unfavorable temperature and pH conditions. It has a sweet sucrose-like taste. The sweetening power of aspartame depends on the nature of the food in which it is incorporated and ranges from 180 to 250 [79]. It is not used in products that are heat-treated. At higher temperatures, the rate of aspartame decomposition increases rapidly [64]; this sensitivity limits the use of aspartame to products that do not undergo intense heat treatment [80]. The taste of aspartame is close to that of sucrose, without major non-specific nuances. Aspartame shows a fairly pronounced synergism in combinations with other sweeteners, with combinations with acesulfame K and saccharin being used most often. It is used in a wide variety of products: soft drinks, puddings, instant drinks, toppings, jams, jellies, cocoa products, dairy products, and chewing gum. The energy intake of aspartame is about 4 kcal/g [81], which is insignificant due to the usual very low concentrations in foods. Aspartame is an excellent sweetener for dehydrated products (powdered drinks and sweetening tablets). At high temperature or low pH, aspartame is gradually broken down with the formation of aspartyl-phenylalanine and methanol [82], which leads to a decrease in the sweetening power of aspartame.

### 2.3. Acesulfame K

Acesulfame K has 200 times the sweetening power of sugar [83]. In the oral cavity, acesulfame K develops a sweet taste that settles quickly and is close to that of sucrose at low concentrations; at higher concentrations, the taste of acesulfame K is affected by a slight residual bitter component, less pronounced than that of sucrose [84]. Acesulfame K has a synergistic effect in combination with other sweeteners; the taste quality is improved in relation to each sweetener individually [85]. Commercially, acesulfame K takes the form of colorless crystals or a white, water-soluble crystalline powder. Acesulfame K is very stable at temperatures of 200 °C and a pH greater than 3, which is why it can be used in the preparation of pastries and confectionery [85,86].

### 2.4. Neohesperidin Dihidrochalcone (NHDC)

Neohesperidine dihydrochalcone (NHDC) is a synthetic sweetener with high sweetening power (1500 and 1800 times stronger than sucrose) [87], with high heat tolerance, and can be used on foods with a long shelf life [88]. NHDC is a naturally occurring bitter-tasting citrus flavanone present in bitter orange fruits (*Citrus aurantium*). Naturally occurring neohesperidine is particularly effective in masking the bitter taste of other compounds found in citrus fruits, including limonin and naringin. Solubility in water at 20 °C is partially soluble but slightly soluble in water at 80 °C [89]. Solubility is also increased in aqueous alkaline solutions and in alcoholic solutions [89]. Also, some polyols (sorbitol) have the property of increasing the solubility of neohesperidine concomitantly with enhancing its flavor. The sweetening power of NHDC is influenced by several factors including: concentration, pH and composition of the product into which it is introduced. The sweetening power of several sweeteners, such as NHDC, can decrease with increasing concentration due to factors like bitterness, and that does not universally apply. Additionally, caffeine can enhance the perception of sweetness in some cases, but not necessarily sweetening power itself. The presence in the food of glucoses, amino acids, or nucleotides causes changes in flavor (sweet, savory, and nutty) in ready-to-eat, starch-based snacks or pre-packed, dry roasted nuts and hazelnuts containing various flavors, E959 is added at 50 mg/kg [89]. In energy-reduced or sugar-free cocoa-based confectionery or dried fruit, neohesperidine is used at 100 mg/kg [90].

### 2.5. Sucralose

Sucralose is a new synthetic sweetener obtained by chemical processes from ordinary sugar. By replacing three “hydroxy” groups in the sucrose structure, a substance with a sweetening power 600 times greater than that of conventional sugar has been obtained [50]. Sucralose is a trichloride derivative of sucrose in the 4, 1′, and 6′ positions. It is a white, odorless, crystalline powder, slightly soluble in water and alcohol [91]. It has good thermal stability and a sweetening power 450 to 700 times that of sucrose, but the sweet taste is affected by a residual component [72]. Because of its high stability at any pH and at high temperatures, sucralose can be used in a wide range of food products with a long shelf life [92]. It is stable at hot and cold temperatures and can be used in cold and hot drinks. Sucralose is useful in baked goods, carbonated drinks, dried dairy products, frozen foods, spreads, and syrups. The taste of sucralose is identical to that of sugar (except for a refreshing, minty tinge) and does not need to be combined with other sweeteners. It shows enhanced synergism in the presence of acesulfame K and cyclamates [72]. It is not recognized in the body as glucose, does not alter blood glucose levels, and passes through the digestive tract unchanged, and the maximum recommended dose is 15 mg/kg body/day [83]. In 2016, following a request from the European Commission, the EFSA Panel ANS concluded that the proposed extension of the use of sucralose in foods for special medical purposes in young children aged 1–3 years would not raise safety concerns [93]. These synthetic sweeteners are continuously monitored by the scientific community, which analyses the safety of sucralose production and consumption [94].

### 2.6. Cyclamate

Cyclamate has a sweetening power 30–50 times that of sucrose [95]. At high concentrations, the sweet taste of cyclamates comes from an unpleasant residual component [12]. The sweet taste occurs after a longer lag phase than sucrose, masking the bitter taste of sucrose when used in a mixture. Cyclamate has good food stability, being stable between pH = 2 and 7 [96]. Commercially, it occurs as colorless crystals or white crystalline powder. It is less commonly used on its own (as mixtures of the two salts of Ca and Na) because of its lower sweetening power, but especially in mixtures with saccharin or aspartame. Cyclamate is tasteless and contains no calories, and when mixed with cyclamate and saccharin, it exhibits a marked synergism, so that a 10:1 ratio results in a pleasantly sweet taste, the cyclamate masking the saccharine taste [97]. Because of its low relative sweetening power, given the maximum doses permitted in foodstuffs, it is necessary to combine cyclamates with other sweeteners. Cyclamate is used in soft drinks, pastries, and fruit processing. In the intestine, only 1% of ingested cyclamate is metabolized; the rest is excreted from the body, so it does not contribute to energy intake through the diet and may have a laxative effect [98].

### 2.7. Alitame

Alitame is a low-calorie sweetener composed of two protein building blocks, L-aspartic acid and D-alanine. Depending on the application, alitamate is about 2000 to 3000 times sweeter than sugar, tastes very similar to sugar, and is cooked and baked [22]. Alitame is a crystalline powder, slightly soluble in water. In the pH range 2–4, characteristic of most carbonated drinks, its stability is 2–3 times better than aspartame [99]. Technologically, it can be used in most food products, including heat-treated foods. It is under evaluation for toxicity. Alitame is not yet approved as a food additive in the European Union [100], but it is in Australia, New Zealand, Mexico, and China, where Alitame is used in table-top confectionery, beverages, dairy products, desserts, bakery products, canned fruit, confectionery, and chewing gum [101].

### 2.8. Neotame

Neotame, a non-caloric artificial sweetener and analogue of aspartame. Chemically neotame is n-[n-[n-(3,3-dimethylbutyl)-l-aspartyl]-l-phenylalanine-1-methyl ester [102]. Sweetening power is 30–40 times greater than aspartame and 7000 to 13,000 times sweeter than sucrose [103]. It has no unpleasant flavors compared to sucrose and enhances original food flavors. It can be used alone or mixed with other sweeteners (aspartame, acesulfame K, sucralose) to increase their individual sweetness and reduce unwanted flavors [47]. It is somewhat more chemically stable than aspartame. Its use can be cost-effective compared to other sweeteners because smaller amounts of neotame are needed. Due to its sweetening power, neotame is suitable for use in carbonated soft drinks, dairy products, cakes, drink powders, and chewing gums. It can be used as a table-top sweetener for hot beverages such as coffee. In 2010, it was approved for use in food in the European Union under the number [102]. It has also been approved as an additive in many other countries outside the US and the EU.

### 2.9. Advantame

Advantame is the newest sweetener to appear on the market since 2008 and has a sweetening power of approximately 20,000–37,000 times that of sucrose [103]. It is a fairly non-caloric, zero-GI artificial sweetener, similar to Neotame, only much sweeter. For many years Neotame held the record of being 8000 times sweeter than sugar, followed by monatin, a natural sweetener with a sweetening power of 3000 times sweeter than sugar [104]. Advantame has been developed by Aijnomoto, a Japanese company that has developed a revolutionary technology to chemically combine aspartame and vanillin, both artificial sweeteners [105]. This new ultra-high potency sweetener offers new opportunities for the food industry. At low concentrations they sweeten, and at ultra-low levels, they act as a flavor enhancer [106].

Advantame is a secondary amine of aspartame and 3-(3-hydroxy-4-methoxyphenyl) propanal (HMPA) [51]. Structurally, advantame resembles a combination of aspartame and vanillin. Advantame shares certain structural characteristics with some natural sweeteners, for example, phyllodulcin. Advantame can be produced by a three-step synthetic chemical process, starting with the production of the main production intermediate, 3-hydroxy-4-methoxycinnamaldehyde (HMCA), from water, sodium hydroxide, and isovanillin in methanol [107]. This is followed by the selective hydrogenation of HMCA to form 3-(3-hydroxy-4-methoxyphenyl) propionaldehydehyde (HMPA). The final step involves N-alkylation of aspartame (L-α-aspartyl-L-phenylalanine methylester) with HMPA to form advantame [103]. Alternatively, high purity HMCA can be obtained externally and thus can be considered the starting material for the production process.

Ingested in the gastrointestinal tract, advantame is hydrolyzed to a carboxylic acid and methanol [53]. In the body, advantame or the acid formed by the hydrolysis is not absorbed, so between 87 and 93% is excreted in the feces and the remainder in the urine [108]. The methanol that is formed is considered harmless due to its small amount. Sensorily, advantame has a clean sweet taste (almost aspartame), with a dominant sweet flavor, with very faint intensities perceived for bitter and sour flavors, it enhances many flavors such as dairy, fruit, citrus, mint, etc. The taste spreads extremely quickly; therefore, it is recommended to be used with another buffer sweetener. It exhibits increased stability at higher temperatures and lower pH values [109]. It does not decompose at high temperatures and thus can be used in all processed foods and cooking. Since 2013, advantame has been approved for use in foods in the EU [110].

### 2.10. Lugduname

Lugduname is an artificial sweetener, with a sweetening power estimated to be between 220,000 and 300,000 times sweeter than sugar, but studies are still ongoing [66]. Lugdunam is part of a family of potent sweeteners containing acetic acid functional groups attached to guanidine [111]. Its use as a sweetener is limited because its toxicity tests have not yet been finalized. The likely toxicity is given by the nitrile groups it contains; therefore, the safe dose in use must be established [32,112]. Artificial sweeteners are the subject of study and evaluation by scientists, and the doses agreed upon by current legislation are safe for the body and the health of the consumer [6].

## 3. Polyols

Polyols, or polyhydric alcohols, are substances obtained by the hydrogenation of carbohydrates (mono-, di-, and oligoglycides) derived from starch, sucrose, and whey: sorbitol, mannitol, maltitol, isomaltose, xylitol, lactitol, and erythritol [112]. Polyols have a sweetening power equal to or less than that of sugar and a spared caloric value of 2–2.5 kcal/g compared to 4 kcal/g for sugar [113]. Natural sweeteners of the polyol type are divided into natural (sorbitol, mannitol, xylitol, fructose) and complex (isomaltose, hydrogenated glucose syrup, lactitol, polydextrose) and are presented in Table 3. Polyols have a number of advantages, such as (i) low caloric intake, giving the product a subtle flavor and a specific texture [114]; (ii) they are slowly absorbed into the body and result in a low glycemic response [115]; and (iii) resistance to the action of bacteria in the oral cavity [116].

**Table 3:** Syrups and other sugar-containing caloric sweeteners.

Sweeteners	Chemical Structure	IUPAC Name	Glycemic Index (GI)	kcal/g	Potency *	References
Golden syrup	C_6_H_12_O_6_	mixture of glucose and fructose, of various ratios	60	3.2	1.1	[117]
Inverted sugar	C_12_H_24_O_12_	(2R,3S,4R,5R)-2,3,4,5,6-pentahydroxyhexanal, and (3S,4R,5S)-1,3,4,5,6-pentahydroxyhexan-2-one	60	4	1.2	[118]
Maltodextrin ^1^	C6nH(10n + 2)O(5n + 1)	(2R,3S,4R,5R)-2,3,4,5,6-pentahydroxyhexanal	110	4	0.01–0.03	[119]
Xylitol	C_5_H_12_O_5_	(2R,3R,4S)-Pentane-1,2,3,4,5-pentol	12	2.4	0.8–1.1	[120]
Sorbitol	C_6_H_14_O_6_	1,2,3,4,5,6-hexanehexol	4	2.6	0.5	[121]
Lactitol	C_12_H_24_O_11_	(2S,3R,4R,5R)-4-[(2S,3R,4S,5R,6R)-3,4,5-trihydroxy-6-(hydroxymethyl) oxan-2-yl] oxyhexane-1,2,3,5,6-pentol	3	2.0	0.4	[122]
Isomalt	C_12_H_24_O_11_	(2R,3R,4R,5R)-6-[(2S,3R,4S,5S,6R)-3,4,5-trihydroxy-6-(hydroxymethyl) oxan-2-yl] oxyhexane-1,2,3,4,5-pentol	2	2.1	0.5	[123]
Mannitol	C_12_H_24_O_11_	hexane-1,2,3,4,5,6-hexol	2	1.6	0.5–0.6	[124]
Maltitol	C_12_H_24_O_11_	4-O-α-D-glucopyranosyl-D-glucitol	35	2.1	0.7–0.9	[105]
Erythritol	C_4_H_10_O_4_	1,2,3,4-butanetetrol	0	0.2	0.65	[125]

* Approximate potencies of high-potency sweeteners (sucrose = 1.0); ^1^—commonly used as a thickener or bulking agent, less as a sweetener.

Technologically, polyols raise a number of problems in use, namely: (i) they are insoluble in water, so must be associated with fat [126]; (ii) most polyols end up undigested in the colon and are subject to the action of bacterial flora [127]; and fermentation produces gases and volatile fatty acids with a laxative effect [128]. Excessive consumption of polyols (over 50 g/day) can cause digestive disorders (diarrhea, bloating) or aggravate irritable bowel syndrome [129].

### 3.1. Xylitol

Xylitol is a sweetening polyol first extracted from birch bark. Xylitol is a natural product, and the human body produces it (about 5–15 mg/day) as part of normal metabolism, as do many animals and plants [130]. It is found in the fibers of many fruits, vegetables, mushrooms, fungi, cereals, or tree bark, where it is produced in small amounts, but the highest natural concentration is in birch bark. Xylitol was discovered simultaneously by researchers in Germany and France (who initially created a xylitol syrup), which was not commercialized until after World War II due to a sugar shortage in Finland [131]. Due to its popularity, increased interest was devoted to studying its behavior in the body, so it was found that it is metabolized in the blood without affecting insulin levels. Regarding the use of xylitol, the code of federal regulations states, “Xylitol may be safely used in foods for special dietary uses, provided that the amount used is not more than is necessary to produce the desired effect” [132]. Due to its health benefits and its applications in food, xylitol is increasingly used. The production of xylitol is realized industrially by chemical, enzymatic, or biotechnological methods [133]. The chemical method of xylitol production is cost inefficient and environmentally unsafe [133]. Biotechnological methods offer cost- and energy-saving opportunities compared to chemical methods. Related technologies involving the use of combinations of enzymes and microorganisms or mixed cultures of microorganisms are the most cost-effective. Thus, various genetic engineering strategies for modulation of important enzymes such as xylose reductase [134], xylozoisomerase [135], and xylosylulokinase [136] for increased xylitol yield and optimization of fermentative parameters based on kinetic studies [137], modelling and simulation are to be used for large-scale xylitol production [138].

### 3.2. Mannitol

Mannitol is a polyol isomer of sorbitol [139]. Mannitol is naturally found in various plant species and produced by microorganisms. In terms of sweetening power, mannitol has about 50% of the sweetening power of sugar (1.6 calories for mannitol with a GI of 2, so it is suitable for diabetics) and has a cooling sensation in the mouth cavity after consumption [140]. In the gastrointestinal tract, mannitol is absorbed in a small amount, so excess consumption may cause gastrointestinal discomfort with a laxative effect [141].

Mannitol is in granular form, has high solubility in aqueous solutions, has high heat stability, and can be used in flavor coatings of various products. Among its applications, mannitol is used as an anti-caking agent due to its minimal ability to absorb moisture from the atmosphere and is suitable for use in products not requiring fermentation. It helps extend the shelf life and texture of food products in the absence of sugar. Industrial production of mannitol is achieved by chemical, enzymatic, and biotechnological methods. For extraction of mannitol from plant sources, supercritical and subcritical fluid technology has been widely used [142]. The chemical synthesis of mannitol involves high-pressure hydrogenation of fructose/glucose mixtures in aqueous solution at high temperature (120–160 °C) with Raney nickel as a catalyst and hydrogen gas [143]. α-Fructose is converted to mannitol, and β-fructose is converted to sorbitol. Glucose is hydrogenated exclusively to sorbitol. Due to the poor selectivity of the nickel catalyst, the hydrogenation of a 50:50 fructose/glucose mixture yields an approximately 25:75 mixture of mannitol and sorbitol. The separation requirement of mannitol and sorbitol results in even higher production costs and low yields [143]. The industrial production of mannitol by fermentation is feasible because a number of homo- and heterofermentative lactic acid bacteria (LAB), yeasts, and filamentous fungi produce mannitol. These bacteria convert fructose to mannitol in 100% yields from a mixture of glucose and fructose (1:2). Glucose is converted to lactic acid and acetic acid, and fructose is converted to mannitol. The enzyme responsible for the conversion of fructose to mannitol is NADPH- or NADH-dependent mannitol dehydrogenase (MDH) [105].

### 3.3. Sorbitol

Sorbitol is a substitute derived from glucose (it is a polyol), naturally present in various fruits and vegetables (red algae, pears, apples, cherries, and peaches), but also produced in normal metabolism. Industrially, it is obtained by chemical synthesis or fermentation. As a sweetener, it has a sweetening power of only 55% of the sweetening power of sugar, a pleasant sweet taste but with a slight aftertaste, a caloric value of 2.6 kcal/g, and a low GI of 4 [144]. It has a low absorption rate in the gut, so consumption of more than 20 g per day can have a strong laxative effect. Commercial sorbitol is a white, crystalline, water-soluble powder commonly used as a bulking agent in various food products [145]. It is a food additive used as a low-calorie sweetener, stabilizer, bulking and thickening agent, and humectant. It is presented as sorbitol or sorbitol syrup [78]. It is often also used as a carrier substance for other ingredients. Sorbitol has the advantage that it does not ferment in the presence of baker’s yeast. Sorbitol is soluble in water, chemically stable, and does not cause crust-soaking during baking.

### 3.4. Maltitol

Maltitol is a non-reducing hygroscopic sugar and disaccharide polyol that is listed as an alternative sweetener to sugar because, except for rumination, it has similar properties to sugar, possesses about 75–90% of the sweetening power of sugar, and has 2.1 calories/gram [51]. From a sensory point of view, maltitol develops a sweet and clean taste with minimal cooling effect in the mouth. It is naturally found in chicory leaves, which contain a small amount, but on a large scale it is obtained by the enzymatic conversion of starch to maltose. Compared to other polyols, it has a higher GI, so it is not as useful for diabetics. Maltitol has food additive status, but the Joint FAO/WHO committee recommends that the “Laxative Threshold Value” (LTV) for maltitol should be a maximum of 60 g per meal [108]. Maltitol is less hygroscopic than sugar and helps maintain moisture in processed foods. This makes it useful when icing or powdering hard-coated candies and chewing gum. It is used in foods, especially in sweet food categories such as cakes, pastries, sugar confectionery, chocolate, chewing gum, and snack bars, and as a table-top sweetener because it has a sweetness similar to sugar (sucrose). A recent study indicates that maltitol does not have significant effects on product quality attributes and might be a suitable alternative for sucrose in “low-sugar” pastry creams [146]. According to the literature, maltitol could be considered as an acceptable substitute for sucrose for the development of 25% lower energy pastry creams and for achieving a lower GI of the products [147].

### 3.5. Lactitol

Lactitol is a polyol, not found as such in nature, and is produced exclusively by catalytic hydrogenation of lactose [148]. Lactitol is a 4-β-d-galactopyranosyl-D-glucitol [149]. Since it is derived from lactose, its use is not recommended for those intolerant to this milk sugar. Lactitol has a sucrose-like molecular weight and a very similar appearance and texture. It tastes good without the presence of an aftertaste. The sweetening power of lactitol is only 40% of the sweetness of sucrose; it has 1.9 kcal/g and a very low GI (only 3 compared to 65 for sugar) [150]. Technologically, it shows high solubility at low temperatures, it is used in mixtures with other sweeteners, it has high sweetening power, and it does not present hygroscopicity, giving baked products shelf stability. Lactitol is stable under both alkaline and acidic conditions. Due to its high solubility in water and its high stability at high temperatures, it is suitable for heat-treated products, especially baked products. Lactitol’s melting point is 146 °C, and it is very soluble in water [151]. It can therefore be used in the quantities specified in recipes in confectionery, pastries, bakery products, preserved fruit products, various desserts based on cocoa, fats, milk, fruit, starch, breakfast cereals, dietary products, and food supplements. Lactitol is fully metabolized and has a beneficial effect on the digestive tract. Sweeteners consumed in moderation might support the growth of bacteria in the colon, which are beneficial to health [151].

## 4. Plant Origin Sweetener’s

Natural sweeteners that are not necessarily carbohydrates have a zero GI and a low or zero calorie content. Research into these natural sweeteners has intensified in recent years as the industry demands a better alternative to synthetic/artificial sweeteners. Table 4 presents several sweetening substances of plant origin with high sweetening power. However, there are plants that have sweetening potential without being classified as carbohydrates and without having significant caloric value, and are described in the following subsections.

**Table 4:** Caloric sweeteners of plant origin.

Sweeteners	Method of Production	Glycemic Index (GI)	Potency *	References
Molasses	mechanical extraction and boiling, followed by a separation process	55	-	[152]
Maple syrup	collecting sap from maple trees and evaporating the water	54	1.0	[153]
Honey	centrifugal extraction	50	1.1	[154]
Sorghum syrup	pressed stalks of sweet sorghum; juice subsequently boiled down	50	1.0	[155]
Sugarcane juice	crystallization, membrane filtration, and solvent extraction	43	-	[156]
Coconut palm sugar	physical indirect heating	35	1	[157]
HFCS-90 (high-fructose corn syrup containing 90% fructose)	corn starch extraction, liquefaction, saccharification, and isomerization, with concern for the degree matters	31	1.6	[158]
HFCS-55		58	1.2	[159]
HFCS-42		68	1.1	[160]
Brown rice syrup	enzymatic hydrolysis of rice starch	25	0.5	[161]
Fructose	acid hydrolysis of inulin or enzymatic conversion of glucose	23	1.17–1.75	[162]
Agave syrup	pressing or shredding the cooked agave to extract the juice, and then concentrating the juice	15	1.5	[163,164,165]

* Approximate potencies of high-potency sweeteners (sucrose = 1.0). “-” high variability.

Sweet taste is detected by taste receptors clustered in the taste buds [6]. Each taste bud presents a set of taste receptor cells, which are receptors that identify molecules and ions present in the food [166] and transduce them into a taste sensation of salty, sour, sweet, bitter, and umami. All sweet molecules, such as carbohydrates, peptides, amino acids, non-nutritive synthetic sweeteners, and sweet-tasting proteins, elicit a sweet taste through the interaction of the sweet taste receptor T1R2-T1R3 and the umami sensor T1R1-T1R3, belonging to the family of G-protein-coupled receptors (GPCRs) [167].

The sweet taste receptor belongs to class C GPCRs, comprising metabotropic glutamate receptors (mGluR), γ-amino butyric acid type B receptors, calcium-sensing receptors (CaSR), and many others [168]. Interaction of sweet taste proteins with the sweet receptor is more difficult to understand because sweeteners are usually low molecular and normally could not be identified [169].

### 4.1. Steviol Glycosides

The plant species *Stevia rebaudiana* has gained attention as a natural alternative to sugar. The *Stevia rebaudiana* plant has 200–300 times the sweetening power [170], zero calories, a low GI [171], and is 480 times sweeter than sucrose.

The leaves of *S. rebaudiana* contain more than 30 different steviol glycosides, among which is rebaudioside A (Reb A). However, stevioside and Reb A exhibit a bitter left and a licorice-like aftertaste, which pose challenges for product formulation. Each steviol glycoside present in the leaf exhibits unique characteristics and different sweetness profiles; for example, Reb A (Rebaudioside A), Reb D (Rebaudioside D), and Reb M (Rebaudioside M)—steviol glycosides—all provide sweetness [36]. In general, all three are pH stable and highly soluble. Individually, these glycosides have distinct flavor profiles, allowing manufacturers to tailor their sweetener blends to meet specific product requirements. Reb A is the most abundant and best-known steviol glycoside, prized for its intense sweetness. Easy to extract, it is widely used because Reb A is about 250–300 times sweeter than sucrose [172].

In the final product Reb A provides an intense sweetness with a slightly lingering aftertaste, especially at high concentrations [36]. It may have a cleaner taste when used in moderation but can contribute to bitterness in larger amounts. Reb D is the best rounded of the steviol glycosides. Reb D offers formulators additional flexibility in creating products with a clean, sugar-like taste and minimal bitterness, having a sweetening power 225–250 times sweeter than sucrose [173]. Reb M is a steviol glycoside naturally present in the stevia leaf and approved fairly recently. It has a sweetening power 225–250 times that of sugar [174], a clean, pleasant flavor, a sucrose-like sweetness profile that comes on quickly. Reb M only occurs in small amounts in conventional stevia plants (less than 0.1%) [175], and this makes it difficult to scale up for large global brands and access for smaller brands.

The starting material for stevia extracts may be natural, so we classify it as a sugar substitute, not an artificial sweetener, but these ingredients are still highly processed. Steviol glycosides are sweeteners obtained by extracting and purifying steviol glycosides from the dried leaves of *Stevia rebaudiana* Bertoni under the action of enzymes. These steviol glycosylated steviol glycosides are then purified by adsorption and ion-exchange chromatography, resulting in a final product containing at least 95% steviol glycosides, comprising a mixture of glycosylated and steviol glycosides [175]. Commercially, stevia extract is found in powder, tablet, or liquid form. Stevia extract is about 200 times sweeter than ordinary sugar, so careful attention should be paid to the amount used. When using the liquid form of the additive, the type of accompanying substance has to be taken into account, as these are considered as additives and usually have maximum permitted levels in the finished product (erythritol) [121]. In the past, EFSA has reviewed the safety of steviol glycosides in terms of stability, their degradation products, metabolism, and toxicity testing [176]. EFSA concluded to set the acceptable daily intake for steviol glycosides, expressed as steviol equivalents, at 4 mg/kg body/day [176]. The rather high demand for Reb M being high, technologies were developed to produce Reb M from stevia leaf extract by bioconversion and fermentation technologies so that fermentation of steviol glycosides, Reb M, and Reb D was produced using yeast. Reb M is approved as an additive in many countries around the world. In 2019, EFSA provided a scientific opinion on the safety of the proposed modification of the specifications for steviol glycosides as a food additive, in particular related to Reb M [176].

### 4.2. Curculin

Curculin, or neoculin, is a complex of sweet proteins that was discovered and isolated in 1990 from the fruit of *Curculigo latifolia* [177]. The curculin has a high sweetening power, approx. 550, and can reach values of over 1000; the sweet taste develops slowly and lasts for several minutes [152]. The interesting thing about this sweetener is that the sweet taste reappears if the subject drinks water. The sweet taste lasts 5 min with water and 10 min with an acid solution [152]. Curculin takes over from the behavior of proteins so that it exhibits a high sensitivity to heat so that at a temperature above 50 °C, the protein starts to degrade and loses its “sweetening” and “taste-modifying” properties [31].

### 4.3. Glycyrrhizin (Liquorice)

Glycyrrhizin is a natural triterpenoid sweetener with a zero GI and a sweetening power of 30–50 compared to sugar [178]. Naturally glycyrrhizin and its salts are found in the root of the shrub *Glycyrrhiza glabra* L. (licorice), from which it is also extracted. The extraction process is relatively simple; the root is boiled, and by concentrating the solution obtained by evaporation, products are obtained both in solid and syrup form. The main active element in licorice is glycyrrhizin, a triterpene saponin 30–50 times sweeter than sugar [70]. Glycyrrhizin is a complex substance with high digestibility and a high solubilization index.

The sweet taste sets in more slowly than with sugar but is much more persistent. However, it exhibits a very intense, almost parasitic sweet taste and a persistent residual aftertaste. In food products, the addition of glycyrrhizin enhances the sweet taste of sucrose and improves the characteristics of other sweeteners such as lactitol or sorbitol. It maintains its licorice flavor characteristics and does not alter the physicochemical characteristics of food products [179]. Glycyrrhizin behaves well in products such as caramels, chocolate, ice cream, chewing gum, and various syrups. In culinary preparations, it is used to mask the salty taste [180]. This natural sweetener is not approved for use in all countries, so national legislation should be consulted before use.

### 4.4. Thaumatin

Thaumatin is a low-calorie sweetener and flavor enhancer. Thaumatin is the common name of a naturally occurring protein blend, consisting of thaumatin I and thaumatin II proteins together with minor amounts of plant constituents, obtained by extraction from the West African katemfe berry, *Thaumatococcus daniellii* Bennett, Thaumatin [181]. Some proteins in the thaumatin family of sweeteners are about 2000 times more potent than sugar and have a persistence of 10–20 min after ingestion [182]. Sensory, thaumatin develops a very sweet, slightly aromatic taste, different from that of sugar, which develops slowly [183]. By combining thaumatin with alanine and some organic acids, the sweetening power is doubled, and it can be used in smaller quantities to achieve the sweetening effect. Thaumatin has the capacity, at concentrations lower than those required for sweetening, to potentiate certain flavors (mint and coffee). Thaumatin is highly soluble in water, stable to heating, and stable under acidic conditions. It is highly soluble in water 600 g/L at 25 °C, stable at 100 °C, pH 5.5, and at room temperature in the range of pH values 2–10 [183]. At pH above 8, thaumatin becomes sensitive to high temperatures; pH control is very important in ensuring stability in food. Thaumatin occurs as an ivory-colored, odorless powder with an intensely sweet taste [184]. Thaumatin is used in confectionery with no added sugar, cocoa products, beverages, dried fruit with no added sugar or low energy value, chewing gum without sweeteners, food ice with no added sugar, in 50 mg/kg [183]. It is authorized in several countries.

### 4.5. Luo Han Guo (Monk Fruit)

Luo Han Guo (*Siraitia grosvenorii* Swingle) is one of the most interesting sweeteners to emerge in recent years. The fruit of Luo Han Guo is very sweet and round and dry, about the size of an orange, and has a thin, brown-colored skin and a pulp containing multiple seeds [185]. These sweet substances account for about 1% of the weight of the fruit and have a sweetening power about 150–200 times that of sugar [186]. Mogrosides consist of a backbone structure called a mogrol with attached glucose units (glycosides). The main mogroside in the monk fruit is mogroside V [187]. It is a natural sweetener with a zero GI, non-caloric, and is suitable for diabetics. Moreover, Monk Fruit sweetener assortments have erythritol as the carrier substance, a substance that needs to be quantified when calculating the erythritol dose per finished product [188]. In the U.S., Luo Han Guo, or monk fruit, is generally recognized as safe (GRAS in FDA terms since 2010) for use as a sweetener or flavor enhancer for food and beverages. In Europe, it has not yet obtained approval by EFSA, which conducts toxicity studies and recommends the use of certain doses in different types of products [189]. Monk fruit-based sweeteners can be added to different foods: beverages such as coffee, hot tea, iced tea, lemonade, and/or smoothies; salads and sauces; soups; yoghurt; oatmeal; or other breakfast cereals. It is marketed as a powder or liquid. Monk fruit granulated or powdered sweetener is the best substitute for sugar because it is the easiest to work with, has a pleasant sweet taste, and is the closest to sugar, especially for icings or toppings. Monk fruit liquid sweetener with a 1:1 erythritol:monk fruit ratio is very popular on the market [190].

### 4.6. Miraculin

Miraculin is a glycoprotein naturally present in the fruit *Synsepalum dulcificum*, also known as the miracle fruit. Miraculin itself is not sweet; in fact, it is not a sweetener per se, but it does have the characteristic of altering the taste for a period of time [191]. Thus, after the taste buds are exposed to miraculin, it binds to sweet taste receptors on the surface of the tongue, and acidic foods that are usually sour (such as citrus fruits) are perceived as sweet, and the effect lasts up to an hour [192]. The detailed mechanism of this taste-inducing behavior is still being studied. It has been suggested that the miraculin protein can change the structure of taste receptors on tongue cells. As a result, sweet receptors are activated by acids, which are generally sour in taste. This effect remains until the taste receptors return to normal.

The sweet effect is influenced by various factors such as miraculin concentration, contact period of miraculin with the tongue surface, and acid concentration. The maximum sweetness-induced response was found to be equivalent to the sweetness of a 17% sucrose solution [192]. While miraculin changes the perception of taste, it does not alter the chemical behavior of food, leaving the oral cavity and stomach mucosa vulnerable to the high acidity of some foods. Miraculin is a thermally unstable substance that is slightly denatured at elevated temperatures, and its activity is inactive at pH below 3 or above 12 at room temperature [54]. It should be noted that the sweet sensation of the fruits is not active when prepared. The glycoprotein is insoluble in water. Keeping the sweet taste of fruits is only possible by freezing. It is used to sweeten drinks, ice cream, and other foods. It is a natural product, contains no calories, and has a zero GI. Commission Regulation (EU) 2021/1974 of 12 November 2021 authorizes the placing on the market of dried fruits of *Synsepalum dulcificum* as a novel food and may be used at a maximum level of consumption of 0.9 g/day, the target population being the general adult population, excluding pregnant and breastfeeding women [193]. The dried fruits of *Synsepalum dulcificum* are safe for consumption by adults when added to food supplements at a maximum daily dose of 0.7 g, equivalent to the dose for an adult person with a standard body weight of 70 kg [193].

### 4.7. Monellin

Monellin is a protein containing 96 amino acids, with a sweet taste, which was discovered in 1969 in the fruit of the West African shrub known as Serendipity Berry (*Dioscoreophyllum cumminsii*) and was first reported as a carbohydrate [194]. The sweetening power of monellin is between 1500 and 2000 times sweeter than sucrose compared to a 7% sucrose solution [195] and 800 times sweeter than sucrose compared to a 5% sucrose solution on a weight basis [152,196]. Sensory analysis indicates that monellin has a clean, high-quality sweet taste without any residual bitterness. Its sweetness develops slowly but persists for an extended duration. Similar to miraculin, the sweetness of monellin is pH-dependent; the protein loses its sweetening properties at a pH below 2 or above 9 [102]. The natural stability of monellin in its original fruit form is notably low; to preserve its sweet taste, the fruit must be stored at temperatures below 20 °C [197]. Heat treatment above 50 °C causes protein denaturation, leading to a loss of sweetness [198]. Nevertheless, monellin remains useful for sweetening protein supplements. Extracting monellin from the fruit is costly because the plant is challenging to cultivate. Industrial production methods, such as chemical synthesis and microbial synthesis, have been developed [152]. For example, monellin has been successfully produced biotechnologically using yeast (*Candida utilis*), which synthesizes this compound [152].

### 4.8. Hernandulcin

Hernandulcin is an intensely sweet sesquiterpene with the molecular formula C_15_H_24_O_2_, a compound that is a constituent of the volatile oil present in the plant *Lippia dulcis* Trev. Subsequent research on hernandulcin identified three structural components responsible for its intense sweet taste: the carbonyl group, the hydroxyl group, and the hydrophobic side chain [199]. Hernandulcin was the first sesquiterpene discovered to have a sweet taste, and after a group of volunteers sampled it, it was determined to be over 1000 times sweeter than sugar [200]. The intensely sweet chemical compound extracted from the plant has a sweetening power ranging from 1000 to 1200 times that of sucrose, although its sweet taste is accompanied by a residual bitter component. Hernandulcin also imparts a refreshing flavor, making it particularly valued for sweetening oral hygiene products [200].

### 4.9. Brazzein

Brazzein is a sweet-tasting protein isolated from the fruit of the climbing plant Oubli *Pentadiplandra brazzeana* Baillon [99]. Two sweet proteins were identified: pentadin (discovered in 1989) and brazzein (discovered in 1994), both highly promising sweetening agents for industrial applications [201]. Brazzein, a protein with sweetening potential, is found in the extracellular pulp surrounding the seeds. Like other sweet proteins, brazzein interacts with taste receptors on the tongue, particularly the T1R3 receptor, which is associated with both sweet and umami taste perception [75]. Brazzein produces a sweet taste with a slight cooling effect in the mouth, similar to liquorice, but with a significantly higher sweetness level. It is estimated to be 500–2000 times sweeter than sucrose by weight [196]. As a protein, brazzein has an energy value of 4 kcal/g [196]. However, due to its intense sweetness, it is used in minute quantities, resulting in a negligible caloric contribution to food products. Technologically, brazzein is heat-stable, remains soluble in water (>50 mg/mL), and maintains its sweetness across a broad pH range (2.5–8) and elevated temperatures [196]. Since the natural brazzein content in *Pentadiplandra brazzeana* fruit is extremely low (0.2%), various heterologous expression systems have been developed for its production [201]. The sweetness of purified brazzein from transgenic tobacco leaves was evaluated by a human taste panel using a double-blind test. Results revealed that brazzein’s sweetening power is approximately 1330 times greater than sucrose by weight, indicating that the protein’s sweetness remains consistent with its native form [202]. The amino acid sequence of brazzein closely resembles that of protease inhibitors found in rapeseed, with similar structural characteristics [152]. The status of brazzein as a sweetener additive remains under regulatory debate. Due to its efficiency, the food industry has shown significant interest in using brazzein in products such as tea, beverages, and chocolate. Despite its potential as a low-calorie sweetener, brazzein has not yet been approved for use as a food additive in the United States or the European Union [194].

### 4.10. Pentadin

Pentadin is a sweet-tasting protein naturally found in the fruits of the *Pentadiplandra brazzeana* Baillon plant. Pentadin is approximately 500 times sweeter than sucrose by weight. It produces a sweet taste with a slow onset and a remarkably prolonged persistence, leaving a lingering sweet/licorice aftertaste at high usage levels [203]. While this sweetener is often overshadowed by brazzein [196], it holds significant potential for use as a standalone sweetening agent. Until then, it may emerge as a valuable by-product of brazzein production.

### 4.11. Phyllodulcin

Phyllodulcin is a natural sweetener; chemically, it is classified as a dihydroisocoumarin, specifically extracted from the leaves of *Hydrangea macrophylla*. Phyllodulcin possesses a sweetening power approximately 400–800 times greater than sucrose solution. Its sweetness develops slowly and is notably persistent [204]. Phyllodulcin is characterized by a bitter sensation, an alcoholic aroma, a cooling effect, and an astringent aftertaste [205]. However, the persistence and onset of sweetness from phyllodulcin are reported to be similar to those of sucrose. The availability of such sweeteners enables the production of specialized food products or low-calorie food items, particularly in the beverage sector.

## 5. Mechanisms of Sweet Taste Perception

**Figure 1:**
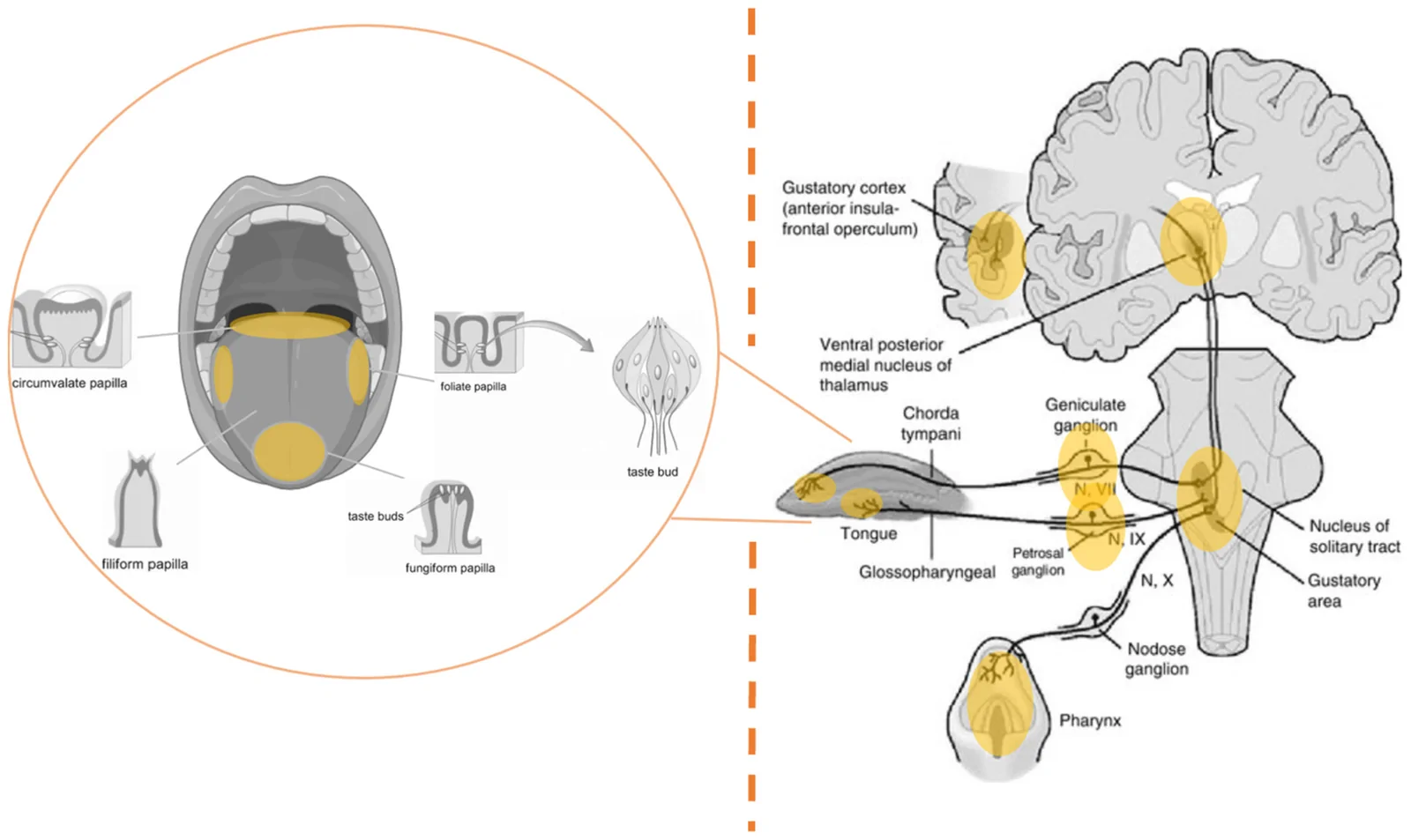
Human mechanism of sweet taste perception, developed and adapted after [206,207].

Sweet taste perception is a complex physiological event involving specialized receptors and neural pathways that interpret and respond to sweet stimuli (Figure 1).

The detection of chemical stimuli, including sweet taste, occurs through specialized taste cells, grouped in taste buds, which are predominantly found on the dorsal surface of the tongue and on the soft palate [208]. Activation of these cells by taste stimuli triggers the release of neurotransmitters to the afferent cranio-cerebral nerve fibers, transmitting taste information to the brain for processing and interpretation. Over the past decade, understanding of the molecular, genetic, and cellular mechanisms of sweet taste detection has increased significantly. Taste receptors belong to the large family of G protein-coupled receptors (GPCRs) [209]. The receptor specific for sweet taste is a heterodimer, meaning it is composed of two distinct subunits: T1R2 and T1R3 [68]. All compounds that evoke a sweet taste bind to and activate this T1R2-T1R3 receptor complex. Sweet taste signalling is mediated not only by T1R2 + T1R3 receptors but also by associated intracellular effectors, including phospholipase CB2 and the transient receptor potential channel MS (TRPMS) [210]. The absence (deletion) of any of these effectors leads to severe deficiencies, if not “taste blindness,” for sweet, umami, and bitter tastes. After activation of the T1R2-T1R3 complex, phospholipase CB2, and TRPMS, depolarization of taste cells and release of neurotransmitters occur. The neural afferents of the cranial nerves then transmit taste information to the rostral division of the solitary tract nucleus (INTS) in the medulla oblongata. In rodents, axonal fibers from the rNTS ascend ipsilaterally to the parabrachial nucleus (PBN) [197], which serves as the second taste relay station. From the PBN, the parallel pathways project to the arvocellular part of the ventroposteromedial nucleus of the thalamus (VPMpc-gustatory thalamic nucleus) and to the amygdala and lateral hypothalamic areas. Thalamic afferents then project to the primary gustatory cortex, located in the insular cortex. in primates, NTS projections appear to bypass the PBN and reach the VPMpc directly. In addition to their presence in the oral cavity, T1R2 and T1R3 receptors are also expressed in enteroendocrine cells in the gastrointestinal tract [167]. There, they may play a role in detecting sugar in the lumen, releasing satiety hormones such as glucagon-like peptide-1 (GLP-1), expressing glucose transporters, and maintaining glucose homeostasis. Genetic variation in T1R genes explains many of the differences observed in the ability to detect sweeteners between species and within the same species. Moreover, while humans perceive aspartame as sweet, rodents are indifferent to it, a result of small differences in the gene encoding T1R2 [42]. Even minor changes in a single amino acid can affect the affinity of the T1R3 subunit for sugars. Also, certain variants of the T2R bitter receptors can react to saccharin [99], providing a molecular explanation for why some people perceive saccharin as both sweet and bitter.

## 6. Comparison Between Natural and Artificial (Synthetic) Sweeteners

**Figure 2:**
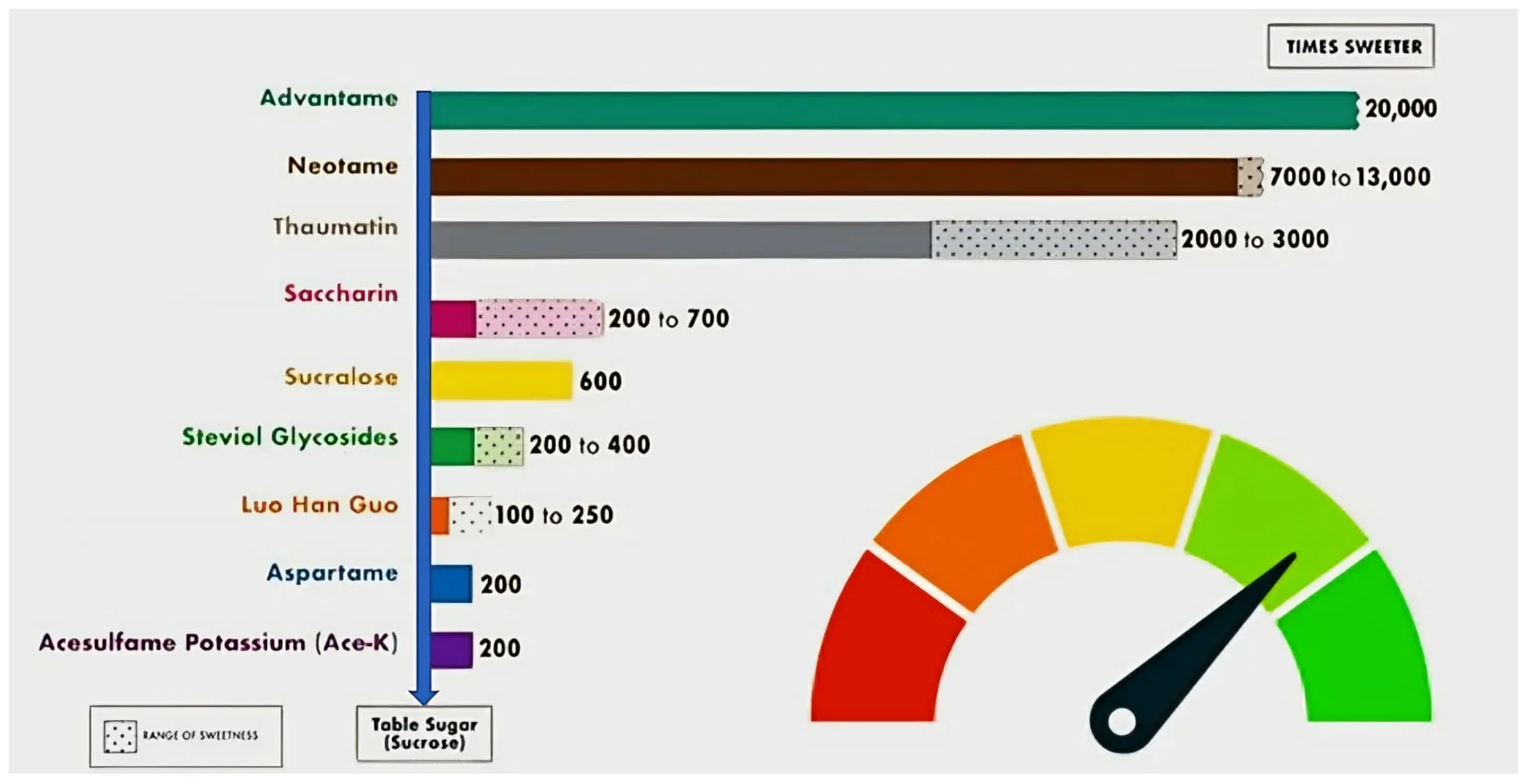
Comparation between sweeteners, after [215,216].

There are two main categories of sweeteners: natural, plant-based sweeteners and artificial or synthetic sweeteners. Natural sweeteners are generally preferred [19,48,177] over synthetic ones because they are not considered to have a negative impact on health. Natural non-sugar sweeteners are low in calories, non-toxic, and much sweeter than sugar (100 to 10,000 times sweeter) [53] (Figure 2), thus overcoming the problems associated with sucrose and synthetic sweeteners [210,211,212,213]. They are useful sugar substitutes for diabetic patients. Many widely used synthetic sweeteners have been found to be carcinogenic and non-nutritive. As a result, the demand for natural non-sugar sweeteners has increased considerably due to their potency, usefulness, safety, and low caloric value as alternatives to sugar. Both nutritive sweeteners (natural, such as sucrose) and NNS (which can be natural or synthetic) activate sweet taste receptors to trigger taste perception in the brain. Although the receptors are the same, different types of sweeteners bind to distinct sites on the T1R2 + T1R3 receptor, as detailed above [167].

There are significant differences between rodents and humans in terms of preferences for certain sweeteners. In addition, at equivalent energy concentrations, humans perceive fructose as sweeter than sucrose, while rats and mice seem to find sucrose sweeter [214]. Rodents show a more limited attraction to NNS compared to humans; rats and mice do not prefer aspartame, and many rats avoid sucralose.

Saccharin is considered a poor substitute for sucrose in rodents [209]. The palatability of sugar is determined not only by its sweet taste but also by its post-oral nutritional effects, which lead to conditioned preference and increased acceptance of flavored solutions associated with intragastric infusions of sugar. This action is not directly mediated by sweet receptors in the intestine (T1R2 + T1R3), as intragastric infusion of sucralose (an NNS) does not condition a flavors preference, even though it activates these receptors [67]. Instead, glucose-specific sensors and the brain’s dopaminergic reward system mediate sugar conditioning. In humans, the taste of NNS sweeteners is not associated with strong satiety signals, unlike concentrated sugar solutions [213].

## 7. Controversies and Limitations of Sweeteners

### 7.1. Health Implications

Sugars, such as mono- and disaccharides, are common components of our diet, providing energy and contributing to flavor and food preservation. The World Health Organization (WHO) recommends limiting the intake of free sugar to less than 10% of total energy intake, with a further reduction to below 5% for additional health benefits [216]. In response to these concerns, non-nutritive sweeteners (NNS), sugar alcohols (polyols), and plant-based sweeteners have emerged as alternatives [217].

**Figure 3:**
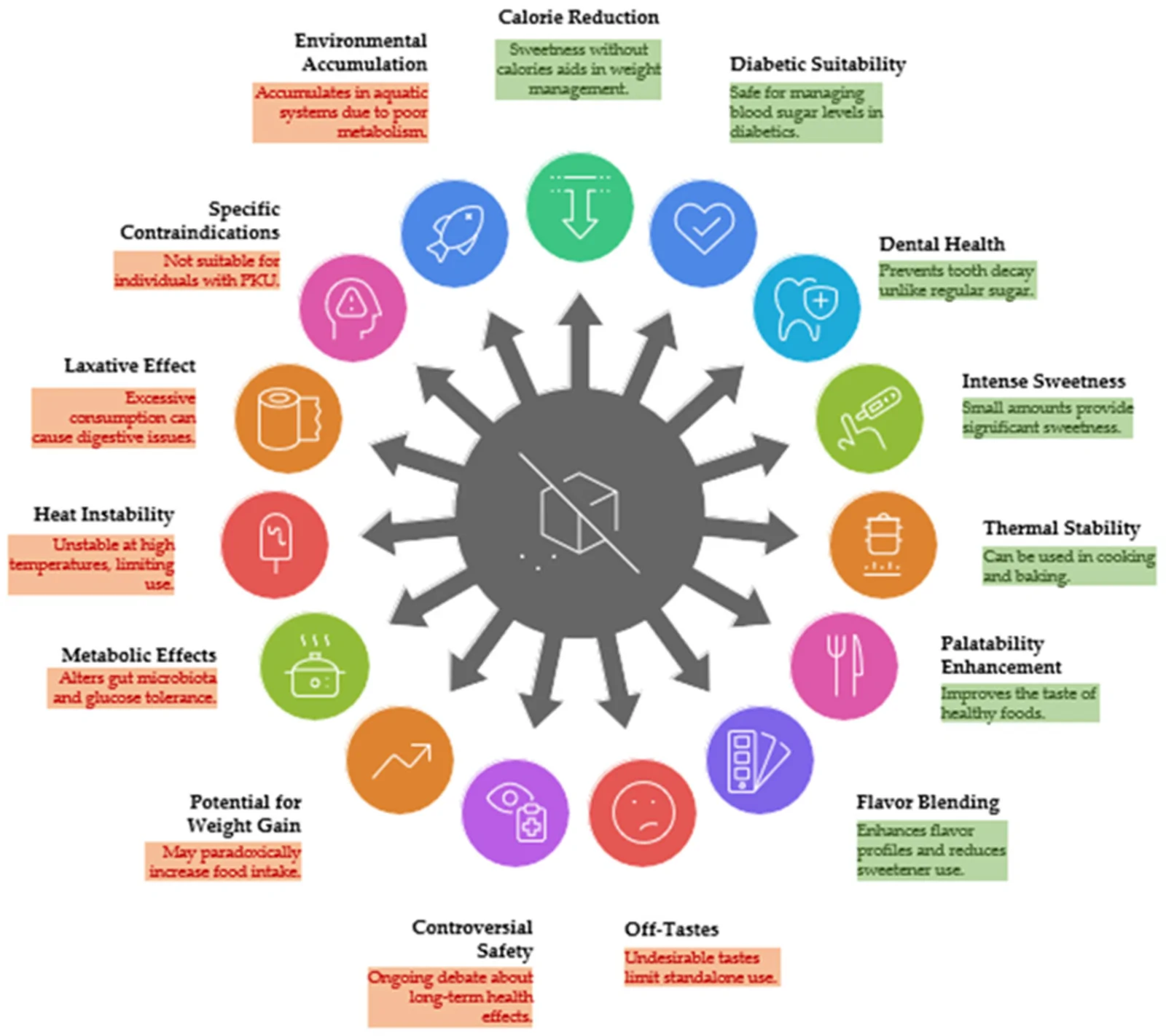
Pros and cons when using sweeteners.

The widespread use of artificial sweeteners [12,17,53,61,72,76,86,181,218,219] has led to considerable debate regarding their health advantages and disadvantages (Figure 3). While regulatory agencies often consider most artificial sweeteners safe [216], stating they are either not metabolized or broken down into naturally occurring components, emerging evidence presents a more complex picture [216]. Some studies suggest potential adverse metabolic effects [219], including an increased risk of weight gain [219], insulin resistance [220], type 2 diabetes [104,173], hypertension [221], and cardiovascular disease [222]. There are also claims linking aspartame to various health problems such as Alzheimer’s disease [104], birth defects, and cancer [221].

A notable discrepancy exists in study outcomes for aspartame’s safety, while other studies report adverse health effects. In July 2023, IARC classified aspartame as “possibly carcinogenic to humans” (Group 2B) based on limited evidence of carcinogenicity in both human and animal studies, reflecting its mandate to identify potential hazards without quantifying actual risk [31]. Although the studies associating sweeteners with cancer may have numerous sources of error, including selection bias, residual confounding, and reverse causality (overweight individuals tend to use these sweeteners and have higher risks of cancer and coronary heart disease).

In contrast, EFSA (2013) and WHO’s Joint Expert Committee on Food Additives (JECFA) (2023) have reaffirmed that aspartame is safe at the established acceptable daily intake (ADI) levels, after evaluating the totality of scientific evidence, including studies cited by IARC, and finding no substantiated carcinogenic effects [222,223]. This distinction underscores the difference between hazard identification, as performed by IARC, and risk assessment, as performed by EFSA and JECFA. Furthermore, IARC’s evaluation was based on a selective subset of studies addressing carcinogenic potential, whereas EFSA and JECFA conducted comprehensive risk assessments integrating all available toxicological, epidemiological, and mechanistic data [104,221,222]. Consequently, while IARC’s classification has elicited public health concern and calls for regulatory caution, the risk-based conclusions of EFSA and JECFA support the continued authorized use of aspartame within current regulatory limits [222,223].

Despite decades on the market and rising consumption, there is a lack of conclusive evidence-based research to definitively discourage or encourage the regular, long-term use of non-nutritional sweeteners (NNS). One animal study using saccharin has shown increased food intake and greater weight gain compared to glucose-sweetened liquids, challenging the notion that NNS aid in weight loss due to calorie deficit [83]. Observational studies in children and adolescents have reported positive correlations between NNS consumption and increased body mass index, though conclusive evidence linking NNS as the cause of weight gain in children is lacking [224,225]. Similarly, a second study found no evidence to link NNS use to weight gain in adults. For optimal health, it is recommended that only minimal amounts of both sugar and NNS be consumed.

The traditional understanding was that NNS did not affect fasting glucose and insulin responses [220]. However, this concept of NNS being metabolically inert is no longer entirely true, as recent research indicates they can be metabolically active [219]. Some studies suggest NNS may impact gut microbiota [55,75,95,141], potentially triggering glucose intolerance [226]. For example, sucralose consumption in rats was linked to a significant decrease in beneficial gut bacteria, weight gain, and altered cytochrome expression, even at low doses approved for human consumption [227]. However, these specific findings were widely criticized for methodological deficiencies. Furthermore, their association is limited because doses higher than ADI are commonly used in animal studies. Human studies have not observed any microbiome effects at 20% of ADI, even at 95% consumption levels (10% ADI only). According to the WHO, to obtain a potentially harmful dose of aspartame, a person should drink more than 14 meal sodas or 80 sweet submarines per day.

One human study reported that saccharin significantly elevated glycemic response during exposure, compared to glucose controls [228]. Conversely, another study has shown no effect of NNS on glucose homeostasis, insulin release, or appetite in healthy individuals or those with type 2 diabetes [229]. For instance, sucralose did not raise blood sugar levels or increase insulin resistance in one study comparing it to sucrose [229]. Artificial sweeteners have also been observed to negatively regulate pathogenic characteristics of certain gut bacteria, such as *E. coli* and *E. faecalis* [230]. A potential explanation for this effect could be related to the biofilm formation and increased bacterial adhesion/invasion into human intestinal cells. It is noted that non-nutritive sweetener consumption may induce person-specific, microbiome-dependent glycemic alterations, necessitating future assessment of clinical implications [104,173,221]. Future long-term studies on human populations are needed to verify the safety of natural sweeteners.

### 7.2. Health and Safety Data

There is ongoing debate regarding the health advantages and disadvantages of artificial and NNS, as regulatory bodies such as the FDA generally endorse their safety [211,221], yet conclusive evidence-based research to strongly encourage or discourage regular use remains lacking, leading to contradictory consumer information [222,223]. Professional organizations, including the Academy of Nutrition and Dietetics, recommend moderation, emphasizing that both sugar and NNS should be consumed sparingly as part of a balanced diet, while individuals are advised to monitor for possible intolerance or allergic responses [231]. In the European Union, the European Food Safety Authority (EFSA) continuously evaluates sweeteners, setting Acceptable Daily Intakes (ADI) based on toxicological data, with ongoing re-evaluations reflecting new evidence [224]. Among individual compounds, aspartame, approved in over 90 countries, has long been considered safe within its ADI (0–40 mg/kg bw) [223], but newer epidemiological studies and animal data have raised concerns about potential associations with certain cancers, prompting its prioritization for re-evaluation by both IARC and JECFA [225]. Moreover, its instability under heat and the production of metabolites such as methanol require careful consideration, and labelling is mandatory due to phenylalanine content [111]. Aspartame is 200 times sweeter than sucrose and provides 4 kcal/g [81,82,111], although its caloric contribution is insignificant due to small required amounts. Aspartame is metabolized into phenylalanine, aspartic acid, and methanol, necessitating a warning label for individuals with phenylketonuria (PKU) [99]. Common reported side effects of aspartame include dizziness, headaches, gastrointestinal issues, and mood changes [232]. While industry-funded studies generally support its safety [217], a number of independently not funded studies suggest adverse health effects [223,224]. It is heat-labile and not ideal for cooking or baking, though its shelf life can be prolonged when blended with more stable NNS like acesulfame-K [82]. Aspartame is commonly found in powdered drink bases (84%), flavored milk drinks (78%), chewing gum (77%), and diet soft drinks (72%) in the German market [76].

Saccharin, recently re-evaluated by EFSA, has have an increased ADI of 9 mg/kg bw [233]; while concerns about genotoxicity from impurities and reproductive toxicity in animal studies remain uncertain [75], human evidence does not strongly support carcinogenic or metabolic risks, although saccharin crosses the placenta and has been detected in fetal tissues [75].

Advantame [104] and miraculin [186], more recently approved sweeteners, show no major safety concerns at authorized levels [234,235], while polyols such as xylitol are generally recognized as safe [114], beneficial for dental health [116], and potentially supportive in cancer care, though excessive consumption may induce gastrointestinal discomfort due to laxative effects. From a broader health perspective, NNS are promoted for obesity and diabetes management, yet evidence remains inconclusive regarding their long-term effects on energy intake, appetite regulation, and cardiometabolic risk, with some studies suggesting neutral or even adverse impacts depending on dose and context. While benefits include reduced caloric load [115], improved dental health [116], and potential niche therapeutic roles, concerns persist around gut microbiota modulation [130], reproductive toxicity in animals, and possible links to cancer in humans. Additional considerations extend to consumer behavior: label reading and awareness of NNS remain low, despite rising demand for transparency, and research shows that interpretive front-of-pack labels (traffic-light systems or health warnings) are more effective in guiding healthier choices than standard nutrition panels [114]. Overall, while NNS provide promising alternatives to reduce sugar-related health risks, their long-term safety profile is complex and context-dependent, requiring cautious consumption, ongoing toxicological reassessment, and improved consumer education to balance potential benefits with emerging risks.

### 7.3. Sensory and Consumers’ Acceptance

The innate human preference for sweetness is a fundamental determinant of food choice, yet increasing awareness of the negative health impacts of excessive sugar intake has driven consumer demand for NNS. In recent years, attention has shifted from synthetic sweeteners to so-called “natural equivalents”, including stevia, monk fruit, erythritol, and allulose, although regulatory definitions of “natural” remain ambiguous [188,189,190].

Despite their potential, NNS frequently exhibit sensory drawbacks compared to sucrose, such as bitterness, metallic undertones, lingering sweetness, or mismatched temporal profiles, which may reduce consumer acceptance [75,84,96,205]. In addition, sucrose replacement can negatively affect other physicochemical and textural properties, such as viscosity, body, and creaminess in beverages or hardness and flavor intensity in jams [236]. The limitations are further compounded by the strong consumer preference for sucrose, particularly in indulgent products like chocolate [237], where taste satisfaction often outweighs health considerations.

On the other hand, NNS offer important advantages, including significant calorie reduction [114], improved glycemic control, and potential benefits for weight management and cardiometabolic health [115], particularly in overweight or obese populations. From a sensory perspective, advances in formulation strategies—such as blending multiple sweeteners or combining them with small amounts of sucrose—can mask off-flavors [61], suppress bitterness [71], and produce temporal sweetness profiles closer to sucrose. For instance, stevia-monk fruit blends enhance sweetness synergy [190], while monosaccharides such as fructose and allulose can mitigate bitter receptor activation induced by certain synthetic sweeteners [57]. Next-generation high-purity steviol glycosides, such as rebaudioside M [36,174], also offer a cleaner sweetness with fewer undesirable side tastes compared to older forms like rebaudioside A [176]. Beyond sensory improvement, some NNSs confer additional product benefits: xylitol supports anthocyanin stability in jams [238], erythritol enhances color intensity [239], and fructose improves brightness and aroma, helping preserve fruit character [240].

Nonetheless, consumer acceptance remains complex and shaped by multiple trade-offs. While the “natural” positioning of certain NNSs appeals to health-conscious consumers, many individuals remain skeptical of artificial sweeteners, and overall awareness and engagement with front-of-package labelling is low.

### 7.4. Process Feasibility and Market Application Trends

From a technological perspective, sweeteners differ significantly in stability: acesulfame-K, sucralose, and saccharin are highly heat and pH stable [70,73,85,86], making them suitable for baking, sterilization, and acidic beverages, while aspartame is unstable at high temperatures and acidic conditions but can be stabilized through encapsulation or blending with more robust compounds [75]. Beyond synthetic options, consumer demand for natural, plant- or fermentation-derived alternatives has accelerated the adoption of sweeteners such as stevia (rich in stevia glycosides), monk fruit (containing mogrosides), xylitol, erythritol, thaumatin, tagatose, brazzein, and allulose [16,114,175,187].

The natural compounds not only provide sweetness but may also deliver additional functional benefits, such as antioxidant properties [6,155,241], anticariogenic effects [238], colour and texture enhancement [239], and favorable impacts on metabolic parameters [115], making them attractive for health-conscious and sustainability-driven markets.

In the context of precision nutrition, the use of artificial and natural non-nutritive sweeteners (NNS) is increasingly viewed not as a universal solution but as part of individualized dietary strategies that account for genetic predispositions [242], metabolic phenotypes, and gut microbiome profiles [243]. Although we do not yet have sufficient knowledge about what constitutes a “healthy” microbiome. Emerging evidence suggests that responses to NNS are highly variable: while some individuals benefit from reductions in caloric intake and improved weight management [244], others may experience altered glucose tolerance or metabolic dysregulation, potentially mediated by host genetics and gut microbial composition [245]. Similarly, gut microbiome diversity has been implicated in modulating glycemic responses to NNS such as saccharin and sucralose, with some microbial communities promoting adverse metabolic effects while others remain neutral [246]. The current variability of NNSs underscores the limitations of population-wide recommendations and highlights the need for precision-based dietary guidance.

Furthermore, metabolomics advancements along with nutrigenomics may guide the tailored dietary interventions, ensuring that the substitution of sugar with NNS maximizes health benefits—such as weight control, reduced glycemic load, and improved oral health—while minimizing potential risks, including metabolic disruption or gastrointestinal discomfort. In this way, precision nutrition could provide a potential framework for optimizing the role of sweeteners in modern diets.

### 7.5. Environmental Sustainability

Various NNS, including saccharin, acesulfame-K, sucralose, and cyclamate, are poorly metabolized by the human body and are excreted unchanged [247]. NNSs reach the environment, primarily the aquatic environment, via wastewater [248]. The ubiquitous occurrence of NNSs in groundwater and surface waters makes them ideal chemical markers of domestic wastewater [249]. The high mobility and relative persistence of NNS residues, and in some cases their transformation products, indicate a likelihood of groundwater contamination [249]. NNS, such as sucralose, acesulfame-K, saccharin, and aspartame, can persist in wastewater, soil, and nectar, raising concerns about their ecological effects on insects that rely on sugars for energy and reproduction [250]. Pollinators such as honeybees (*Apis mellifera*) and bumblebees (*Bombus* spp.) may experience altered foraging behavior, reduced learning and memory, or disruptions in gut microbiota when exposed to NNS-contaminated nectar [249], potentially affecting colony growth and pollination services. Other floral-visiting insects, including butterflies, moths, and hoverflies, may have reduced feeding efficiency or altered visitation patterns, while sugar-feeding ants could show changes in food preference and colony development [249]. Emerging effects arise primarily through interference with chemosensory perception, energy metabolism, and gut microbiota composition [249], which in turn can cascade to broader ecosystem impacts, including diminished pollination and altered plant-insect interactions. Environmental entry of NNS occurs via wastewater discharge, agricultural runoff, and food-processing residues, highlighting a need for monitoring and sustainable management [246].

Integrating ecological considerations with human dietary use of sweeteners emphasizes the importance of developing biodegradable, low-impact alternatives; optimizing sweetener formulations; and promoting targeted environmental stewardship to protect insect populations and maintain ecosystem stability.

### 7.6. Commercialisation Challenges

The global artificial sweetener market is significant, valued at USD 2.9 billion in 2024 and projected to grow from USD 3.11 billion in 2025 to USD 5.44 billion by 2033, with a Compound Annual Growth Rate (CAGR) of 7.25% during this forecast period, while another source estimates the market value at USD 10.5 billion in 2025, forecasted to reach USD 15.2 billion by 2035 at a CAGR of 2.9% [251]. Despite the growing demand, the artificial sweetener market faces several commercialization challenges, including ongoing debate over their health effects [217,221,222,223], as some studies have linked them to potential risks such as cancer, obesity, and other illnesses.

The marketplace for artificial sweeteners is characterized by robust growth drivers, dominant product types, end-user segments, and key regional strongholds [252]. Drivers include the increasing prevalence of lifestyle diseases like diabetes and obesity, as over 80% of diabetics globally reside in low- and middle-income nations, where the disease prevalence is expected to rise, boosting demand [253].

By shifting the consumer preferences toward healthier options, as evidenced by 40% of American consumers reducing sugar intake in the past year [252,253,254]. Furthermore, 65% of global consumers are decreasing sugary beverage consumption [255]. In addition, urbanization and changing dietary habits are leading to higher demand for convenient, low-calorie foods, with 35% of diabetics worldwide substituting artificial sweeteners for sugar in 2025 [256]. However, the food and beverage industry is adapting by incorporating artificial sweeteners into condiments, sodas, desserts, snacks, and other products, while investing in R&D, distribution contracts, mergers, facility expansions, and advertising to maintain competitiveness. In terms of market structure, aspartame dominates, holding a 50% value share in 2025 due to its high sweetness intensity, sugar-like flavor profile, versatility, stability across pH and temperature ranges, and affordability [257], with other significant sweeteners including Ace-K, sucralose, saccharin, and cyclamate. Powder forms dominate the market due to ease of handling, storage, and precise measurement, while liquid forms are preferred for beverages and syrups.

North America is the largest market, projected to grow at a CAGR of 6.8% [251], driven by health-conscious consumers, high obesity and diabetes prevalence, and strict sugar content regulations. Following, China shows a projected CAGR of 3.2% through 2035 due to rising diabetes and obesity rates and increasing health awareness [251]. The UK is expected to hold a 30% global market share due to regulatory initiatives and consumer awareness, with Europe overall estimated to grow at a CAGR of 7.35% [251]. Market concentration includes tiered companies, with Tier 1 leaders holding 40% market share, Tier 2 mid-size players holding 50%, and Tier 3 small-scale businesses holding 10%, with leading manufacturers including DuPont, Tate & Lyle PLC, Cargill, Archer Daniels Midland, Ajinomoto, Nestlé, Ingredion, Wilmar, JK Sucralose, and Roquette Freres [258]. Overall, the artificial sweetener market reflects a dynamic interplay between rising health-driven demand and persistent regulatory, scientific, and consumer acceptance challenges, shaping its trajectory as both an opportunity and a controversy within the global food industry. A potential trend in novel developments is to include plant-based, clean-label stevia solutions in order to mimic sugar’s sensory properties without additives.

## 8. Conclusions

In conclusion, the sugar-reduction trend is essential for public health, necessitating the food industry’s exploration of viable alternatives. Sugar replacement is complex, given its multiple roles in products. A wide range of artificial sweeteners, polyols, and natural sweeteners is available, each with distinct sensory and metabolic properties. These offer benefits such as reduced calories and minimal glycemic impact but require careful selection due to potential digestive side effects or residual taste. Consumer acceptance and adoption vary, though all approved alternatives are rigorously studied and regulated. Ongoing research and monitoring of sweetener safety remain mandatory to optimize their availability for industrial applications. Notably, recent studies investigating the effects of engineered sweeteners on the human gut microbiota have produced conflicting results, with some reports suggesting potential alterations in microbial composition and glucose metabolism, while others indicate negligible effects. In addition, it is important to acknowledge the ongoing controversy regarding the potential link between artificial sweeteners and cancer. Much of this debate stems from animal studies conducted with doses far exceeding the Acceptable Daily Intake (ADI), which may not accurately reflect realistic human exposure. Nevertheless, these findings have raised public concern and continue to stimulate further research into the long-term safety of high-dose consumption. This highlights the need for further long-term research, particularly in specific populations such as patients with diabetes mellitus, to clarify the interplay between sweetener consumption, gut microbiota, and metabolic health.

## Data Availability

No new data were created or analyzed in this study. Data sharing does not apply to this article.
