# Correction: Dragomir et al. Beyond Sugar: A Holistic Review of Sweeteners and Their Role in Modern Nutrition. *Foods* 2025, *14*, 3182

**DOI:** 10.3390/foods15101779

**Published:** 2026-05-18

**Authors:** Nela Dragomir, Daniela-Mihaela Grigore, Elena Narcisa Pogurschi

**Affiliations:** Faculty of Animal Productions Engineering and Management, University of Agronomic Sciences and Veterinary Medicine of Bucharest, 59 Marasti Blvd, District 1, 011464 Bucharest, Romania; nela.dragomir@usamv.ro (N.D.); elena.pogurschi@usamv.ro (E.N.P.)

## 1. Error in Table

In the original publication [1], there were some mistakes in Table 1. We modified the caption for Table 1 and have changed the column header from sweetener to mono- and disaccharides, in order to fit the description. The type of treatments for sucrose has been updated to extracted and purified through a series of physical and chemical operations. The type of treatments for galactose has been updated to hydrolysis of lactose or microbial fermentation. Additions have been made for the missing values for fructose GI, 23, and the range of potency 1.17–1.75. We corrected the spelling of “tagatose” in Table 1. The molecular formula for isomaltose in Table 1 has been updated. The corrected Table 1 appears below.

There was a mistake in Table 2. We modified the caption in order to add the Lugduname sweetener and its potency. The corrected Table 2 appears below. With this correction, the order of some references has been adjusted accordingly.

There was a mistake in Table 3. Caramel should not be listed in Table 3, as it is a coloring agent and not a sweetener. The IUPAC name for inverted sugar has been updated to (2R,3S,4R,5R)-2,3,4,5,6-pentahydroxyhexanal, and (3S,4R,5S)-1,3,4,5,6-pentahydroxyhexan-2-one. The IUPAC name for maltodextrin has been updated to (2R,3S,4R,5R)-2,3,4,5,6-pentahydroxyhexanal. The IUPAC name for xylitol has been updated to (2R,3R,4S)-pentane-1,2,3,4,5-pentol. With this correction, the original citation of reference [117] in Table 3 has been deleted. The order of some references has been adjusted accordingly. The corrected Table 3 appears below. With this correction, the order of some references has been adjusted accordingly.

There was a mistake in Table 4. We corrected the molasses term. The corrected Table 4 appears below.

## 2. Text Correction

A correction has been made to the abstract, 220,0000 should be 220,000.

A correction has been made to Section 2, Section 2.6. The third-to-last sentence in Section 2.6 has been deleted.

A correction has been made to Section 4. We adjusted the final proposition of the first paragraph of the 4th section in order to clarify and introduce Section 4 information. The corrected sentence should read: “Table 4 presents several sweetening substances of plant origin with high sweetening power. However, there are plants that have sweetening potential without being classified as carbohydrates and without having significant caloric value, and are described in the following subsections.”.

A correction has been made to the caption of Figure 1. We added content in order to clarify the inspiration for Figure 1. The updated caption of Figure 1 is “Figure 1. Human mechanism of sweet taste perception, developed and adapted after [206,207].”.

A correction has been made to Section 7, Section 7.5, paragraph 1. The mention of Utetheisa Kong white has been removed from sentence 7. The original citations of references [251,252] have been deleted. With this correction, the order of some references has been adjusted accordingly. The corrected sentences should read: “Pollinators such as honeybees (*Apis mellifera*) and bumblebees (*Bombus* spp.) may experience altered foraging behavior, reduced learning and memory, or disruptions in gut microbiota when exposed to NNS-contaminated nectar [249], potentially affecting colony growth and pollination services. Other floral-visiting insects, including butterflies, moths, and hoverflies, may have reduced feeding efficiency or altered visitation patterns, while sugar-feeding ants could show changes in food preference and colony development [249]. Emerging effects arise primarily through interference with chemosensory perception, energy metabolism, and gut microbiota composition [249], which in turn can cascade to broader ecosystem impacts, including diminished pollination and altered plant-insect interactions.”

The authors state that the scientific conclusions are unaffected. This correction was approved by the Academic Editor. The original publication has also been updated.

## Figures and Tables

**Table 1:** Common sweeteners and their Glycemic Index (GI).

Mono- and Disaccharides	Type of Treatments	Chemical Formulas	Glycemic Index (GI)	Potency *	References
Maltose	enzyme-catalyzed hydrolysis	C_12_H_22_O_11_	105	0.3	[8]
Dextrose	hydrolysis	C_6_H_12_O_6_	100	0.7	[9]
Glucose	hydrolysis	C_6_H_12_O_6_	100		[10]
Trehalose	enzymatic and/or fermentation processes	C_12_H_22_O_11_	70	0.4–0.5	[11]
Sucrose	extracted and purified through a series of physical and chemical operations	C_12_H_22_O_11_	65	1.0	[12]
Fructose	enzymatic conversion of glucose	C_6_H_12_O_6_	23	1.17–1.75	[13]
Galactose	hydrolysis of lactose or microbial fermentation	C_6_H_12_O_6_	20	0.5–0.7	[14]
Lactose	concentration, crystallization, and purification from dairy	C_12_H_22_O_11_	46	0.2–0.4	[15]
Tagatose	isomerization of D-galactose	C_6_H_12_O_6_	1–3	0.9	[16]
Isomaltose	starch hydrolyzation	C_12_H_22_O_11_	35	3.5	[17]

* Approximate potencies of high-potency sweeteners (sucrose = 1.0).

**Table 2:** Artificial sweeteners potency.

Sweeteners ^1^	Potency *	References
Advantame	20,000	[10]
Acesulfame-K	200	[58]
Alitame	2000	[59]
Aspartame	180	[60]
Cyclamate	30–40	[61]
Neotame	8000	[62]
Neohesperidin dihidrochalcon (NHDC)	1500–1800	[63]
Saccharin	300	[64]
Sucralose	600	[65]
Lugduname	220,000	[66]

* Approximate potencies of high-potency sweeteners (sucrose = 1.0); ^1^ all sweeteners are 0 kcal/g, except for aspartame, 4 kcal/g.

**Table 3:** Syrups and other sugar-containing caloric sweeteners.

Sweeteners	Chemical Structure	IUPAC Name	Glycemic Index (GI)	kcal/g	Potency *	References
Golden syrup	C_6_H_12_O_6_	mixture of glucose and fructose, of various ratios	60	3.2	1.1	[117]
Inverted sugar	C_12_H_24_O_12_	(2R,3S,4R,5R)-2,3,4,5,6-pentahydroxyhexanal, and (3S,4R,5S)-1,3,4,5,6-pentahydroxyhexan-2-one	60	4	1.2	[118]
Maltodextrin ^1^	C6nH(10n + 2)O(5n + 1)	(2R,3S,4R,5R)-2,3,4,5,6-pentahydroxyhexanal	110	4	0.01–0.03	[119]
Xylitol	C_5_H_12_O_5_	(2R,3R,4S)-Pentane-1,2,3,4,5-pentol	12	2.4	0.8–1.1	[120]
Sorbitol	C_6_H_14_O_6_	1,2,3,4,5,6-hexanehexol	4	2.6	0.5	[121]
Lactitol	C_12_H_24_O_11_	(2S,3R,4R,5R)-4-[(2S,3R,4S,5R,6R)-3,4,5-trihydroxy-6-(hydroxymethyl) oxan-2-yl] oxyhexane-1,2,3,5,6-pentol	3	2.0	0.4	[122]
Isomalt	C_12_H_24_O_11_	(2R,3R,4R,5R)-6-[(2S,3R,4S,5S,6R)-3,4,5-trihydroxy-6-(hydroxymethyl) oxan-2-yl] oxyhexane-1,2,3,4,5-pentol	2	2.1	0.5	[123]
Mannitol	C_12_H_24_O_11_	hexane-1,2,3,4,5,6-hexol	2	1.6	0.5–0.6	[124]
Maltitol	C_12_H_24_O_11_	4-O-α-D-glucopyranosyl-D-glucitol	35	2.1	0.7–0.9	[105]
Erythritol	C_4_H_10_O_4_	1,2,3,4-butanetetrol	0	0.2	0.65	[125]

* Approximate potencies of high-potency sweeteners (sucrose = 1.0); ^1^—commonly used as a thickener or bulking agent, less as a sweetener.

**Table 4:** Caloric sweeteners of plant origin.

Sweeteners	Method of Production	Glycemic Index (GI)	Potency *	References
Molasses	mechanical extraction and boiling, followed by a separation process	55	-	[152]
Maple syrup	collecting sap from maple trees and evaporating the water	54	1.0	[153]
Honey	centrifugal extraction	50	1.1	[154]
Sorghum syrup	pressed stalks of sweet sorghum; juice subsequently boiled down	50	1.0	[155]
Sugarcane juice	crystallization, membrane filtration, and solvent extraction	43	-	[156]
Coconut palm sugar	physical indirect heating	35	1	[157]
HFCS-90 (high-fructose corn syrup containing 90% fructose)	corn starch extraction, liquefaction, saccharification, and isomerization, with concern for the degree matters	31	1.6	[158]
HFCS-55		58	1.2	[159]
HFCS-42		68	1.1	[160]
Brown rice syrup	enzymatic hydrolysis of rice starch	25	0.5	[161]
Fructose	acid hydrolysis of inulin or enzymatic conversion of glucose	23	1.17–1.75	[162]
Agave syrup	pressing or shredding the cooked agave to extract the juice, and then concentrating the juice	15	1.5	[163–165]

* Approximate potencies of high-potency sweeteners (sucrose = 1.0). “-” high variability.
